# Electrochemical Disposable Biosensor to Monitor Dabigatran in Point-of-Care Anticoagulation Therapy

**DOI:** 10.3390/molecules28134953

**Published:** 2023-06-23

**Authors:** Ashwin K. V. Mruthunjaya, Ronald C. Chatelier, Angel A. J. Torriero

**Affiliations:** School of Life and Environmental Sciences, Deakin University, Burwood, VIC 3125, Australia

**Keywords:** dabigatran, electrochemical sensor, point-of-care

## Abstract

Dabigatran etexilate, an oral prodrug, is often used to treat complications linked to thrombosis. Dabigatran (DAB, active form) does not need to be monitored. However, there are several conditions, such as reduced renal function, traumatic bleeding, emergency surgery, the need for thrombolytic therapy in acute stroke, or the requirement to use other forms of anticoagulation, where knowing the concentration of DAB in the blood is indispensable. Unfortunately, there are no convenient DAB-specific point-of-care tests available. To solve this problem, two disposable sensors were constructed and optimised in this work to detect the anticoagulant drug DAB using novel co-facing disposable electrodes, which allows a calibration-free quantitation of the electroactive mediator concentration. A trypsin-based sensor was evaluated. This sensor performed well in a 10 mM Tris buffer (pH 8.8) solution. However, trypsin was inhibited by alpha-1 antitrypsin when a plasma sample was introduced into the sensor. This problem was overcome by plasma filtration. This sensor showed a detection limit of 50.7 ng mL^−1^ DAB in plasma and a quantification range of 177–500 ng mL^−1^. A thrombin-based sensor was also constructed. This sensor performed well in ten-fold diluted plasma, overcoming the filtration problem observed with the trypsin-based sensor. This sensor showed a detection limit of 9.6 ng mL^−1^ DAB in plasma and a quantification range of 11.5–140 ng mL^−1^. Its extensive pH stability range, the possibility of working at physiological pH, low volume, low cost, and fast turnaround response (less than 20 s) make the calibration-free thrombin-based sensor a suitable point-of-care test to measure DAB concentration in the blood.

## 1. Introduction

**Scheme 1:**
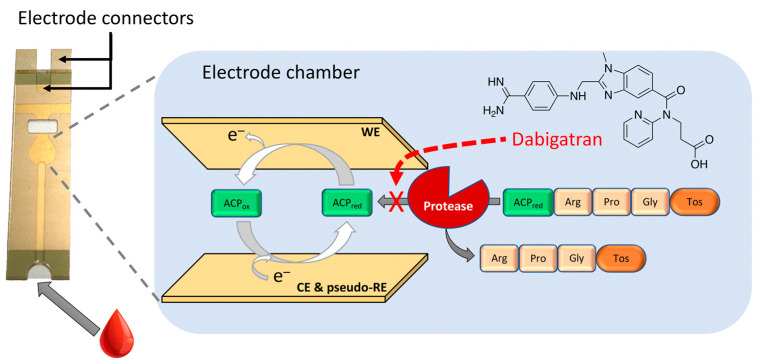
Photo of the disposable sensor (left) together with a schematic representation of the enzymatic reaction followed by an electrochemical signal amplification inside the disposable co-facing gold electrode.

Thromboembolism-related complications such as myocardial infarction, stroke, and deep vein thrombosis are frequent causes of death and disability worldwide [1,2]. Traditionally, warfarin and heparin have been used to prevent and treat these diseases. More recently, newer agents known as direct oral anticoagulants have been introduced. Dabigatran etexilate, an oral prodrug, is an example of this new group. In its active form, dabigatran (DAB, Scheme 1) directly and competitively inhibits free and clot-bound thrombin (a serine protease part of the coagulation cascade), offering critical clinical advantages over warfarin. These advantages include predictable pharmacokinetic and pharmacodynamic effects, minimal medication and food interactions, and fixed dosing without requiring routine laboratory monitoring of coagulation status [3,4,5]. Despite this, there are several conditions, such as reduced renal function, traumatic bleeding, emergency surgery, the need for thrombolytic therapy in acute stroke, or the requirement to use other forms of anticoagulation, where knowing the concentration of DAB in the blood is indispensable [6].

Currently, no DAB-specific point-of-care tests are available to measure the concentration of the drug in particular conditions of massive bleeding [7]. The traditional coagulation assays, such as the international normalised ratio, prothrombin time, activated partial thromboplastin time, and diluted thrombin time tests, are insensitive to most direct oral anticoagulants, including DAB [6,8]. The diluted thrombin time-based assays can quantify dabigatran at relatively low levels. However, these results are unreliable in the case of massive overdose [6]. A liquid chromatography-tandem mass spectrometry technique and an anti-dabigatran enzyme-linked immunosorbent assay (quantification range 7.8–125 ng mL^−1^) were developed [9,10,11]. However, these laboratory techniques cannot be used at the point-of-care.

DAB is a benzamidine-based trisubstituted benzimidazole derivative [12]. The active benzamidine moiety forms a salt bridge with the carboxylate of the thrombin aspartate residue Asp-189, inhibiting the enzyme activity [12]. Besides, DAB can also inhibit trypsin’s enzymatic activities. The inhibitor constants for human thrombin and trypsin are 4.5 ± 0.2 and 50.3 ± 0.3 nM, respectively [12]. Therefore, this property can be used to develop a point-of-care sensor. For example, different colourimetric, fluorescent and electrochemical detection systems were developed to monitor the protease activity [13,14,15,16,17,18], being the electrochemical technique studied more extensively due to its low cost, simplicity, reduced size, and high sensitivity.

The different surface-confined electrochemical approaches used to determine protease activity were as follows [19]:

1. the proteolytic removal of a blocking layer on an electrode, such as a gelatine film and a charged oligopeptide monolayer,
2. the proteolytic removal of an electroactive species such as ferrocene, methylene blue, or 4-aminodiphenylamine, attached to an electrode,
3. the proteolytic generation of an electroactive species such as 4-nitroaniline and 4-amino-2-chlorophenol, and
4. the proteolytic conversion of a polyionic polypeptide into fragmented amino acids.

The requirement to add a film or monolayer to the sensing electrodes is a significant drawback of these techniques. Using an incubation period of 120 or 30 min, Park and Yang modified option 3 to create a solution-based trypsin sensing technique with a detection limit of 1 and 100 ng mL^−1^, respectively [19].

Redox catalysis and electric current enhancement due to the cycling of an electroactive reversible molecule are practical approaches to improve the detection limit and reduce the analysis time [19,20]. Consequently, using the previously mentioned option 3 followed by an efficient redox cycling approach should produce an optimal response in a considerably short time. Examples of similar systems are the use of electrochemical redox cycling, electrochemical–enzymatic, electrochemical–chemical, and electrochemical–chemical–chemical redox cycling [19,21,22,23,24]. In the present work, DAB electrochemical biosensors with enhancement by redox cycling are reported, resulting in a simple, fast, cheap, and sensitive method based on the inhibition properties of DAB on trypsin and thrombin combined with the amplification properties obtained with the use of novel co-facing disposable electrodes [25,26,27]. The theory and characterisation of these sensors were previously presented by our group [27]. Besides, this sensor platform has been successfully applied to glucose monitoring, with more than 10 billion OneTouch^®^ Verio^®^ strips sold by LifeScan Inc. It also was applied to the coagulation testing (Xprecia Stride^TM^ PT/INR sensor, Siemens Healthineers and Universal Biosensors) and wine sensors for free SO_2_, malic acid, acetic acid, fructose, glucose, or total titratable acidity (Sentia^TM^ range sensors, Universal Biosensors) [28]. The measuring principle of this biosensor for detecting DAB is shown in Scheme 1. In the presence of the Tos-Gly-Pro-Arg-ACP peptide, the proteases trypsin and thrombin catalyse the release of the redox-active 4-amino-2-chlorophenol (ACP). However, when DAB is present, it readily inactivates the proteolytic enzymes and decreases the amperometric current measured. Experimental parameters, such as applied potential, substrate concentration, and interferences in plasma samples, were optimised to obtain effective electrochemical responses. The resulting assay exhibits greatly enhanced selectivity and allows the sensitive electrochemical detection of DAB. The results presented in this work open a new avenue to achieve a simple and efficient amplification reaction, which is potentially valuable for many point-of-care applications.

## 2. Results and Discussion

### 2.1. Voltammetric Study of Tos-Gly-Pro-Arg-ACP

**Figure 1:**
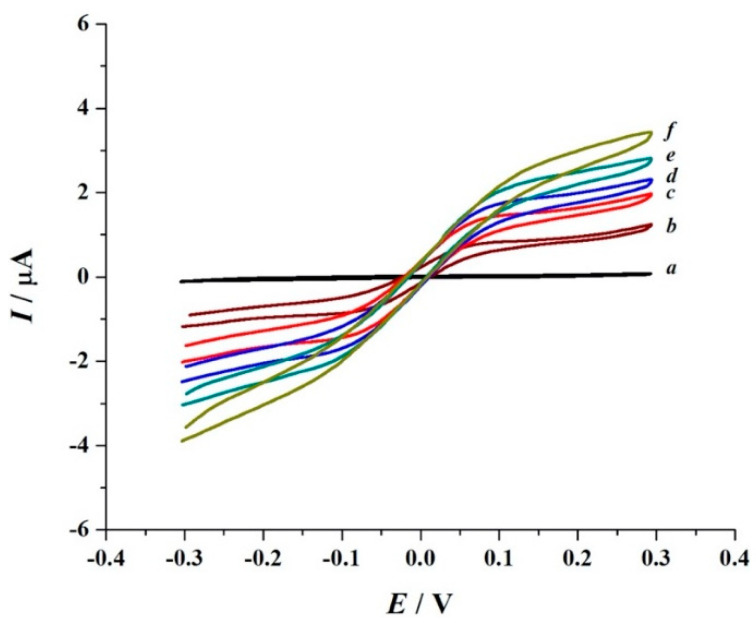
Cyclic voltammograms of 1.0 mM Tos-Gly-Pro-Arg-ACP in 20 mM Tris buffer (pH 8.8) (*a*) in the absence and (*b*–*f*) presence of 0.10 µM trypsin obtained with the use of the disposable co-facing gold electrodes. Voltammograms (*b*–*f*) were obtained within 4 min after loading the mixture onto the disposable electrode. Scan rate: 0.05 V s^−1^; T: 37 ± 0.2 °C.

Figure 1-curve *a* shows the voltammogram of 1.0 mM Tos-Gly-Pro-Arg-ACP in 20 mM Tris buffer (pH 8.8) obtained using the co-facing two-electrode system (Scheme 1), where one electrode act as the working electrode and the second one as both the counter and the pseudo-reference electrode. The Tos-Gly-Pro-Arg-ACP substrate contains a protective tosyl (Tos) group coupled to the N-terminal and an ACP group connected to the C-terminal of the tripeptide. The ACP group is not electroactive under this condition, as the nitrogen lone pair of electrons required to produce the oxidation product 2-chloro-*p*-quinoneimine (ACP_ox_) is involved in the Arg-ACP amide bond. Consequently, no faradaic current was observed within the potential window studied.

The oxidation of ACP (obtained as a product of the enzyme catalytic action on Tos-Gly-Pro-Arg-ACP) to the corresponding ACP_ox_ was also studied. Figure 1-Curves *b*–*f* provides examples of the reversible ACP voltammograms observed upon adding a mixture of 1.0 mM Tos-Gly-Pro-Arg-ACP and 0.10 µM trypsin in 20 mM Tris buffer (pH 8.8) to the chamber formed by the two co-faced electrodes. The addition of trypsin triggers the appearance of reversible sigmoidal-shaped curves, which magnitude is a function of the enzymatic catalytic activity. This is represented in Figure 1 by consecutive voltammograms with increased limiting current obtained at different times during the first four minutes after mixing the enzyme and substrate. The half-wave Potential, *E*_1/2_, for the sigmoidal curve, is equal to 0.01 ± 0.03 V vs. Au pseudo-reference electrode and independent of the ACP concentration and potential scan rate used. This *E*_1/2_ is equivalent to the *E*_m_ (calculated from the average of the oxidation (*E*_p_^ox^) and reduction (*E*_p_^red^) peak potentials (*E*_m_ = 1/2(*E*_p_^ox^ + *E*_p_^red^)) [29,30], obtained for transient voltammogram and is highly reproduced in all disposable electrodes used (n = 50).

The applied potential for the optimal chronoamperometric (CA) current response is obtained where the limiting current dominates the voltammetric behaviour. Based on Figure 1, any potential value ≥0.20 V vs. Au pseudo-reference electrode should fulfil this requirement. Therefore, considering the response sensitivity and operational stability, the 0.40 V vs. Au pseudo reference electrode was selected as the applied potential for ACP concentration change monitoring.

The effect of impurities in the CA background current introduced by the redox specie present in excess, [Fe(EDTA)]^−1^, has been previously documented to introduce up to 15% error in the measurements [27]. Therefore, background subtraction is required before the analysis of the respective CAs. This procedure also eliminates the interference of any redox active molecule present in the plasma sample used, increasing the selectivity of this sensor.

For the optimisation of different assay parameters, such as pH and substrate concentration, the current at 60 s was initially used.

### 2.2. Optimisation of pH and Substrate Concentration

**Figure 2:**
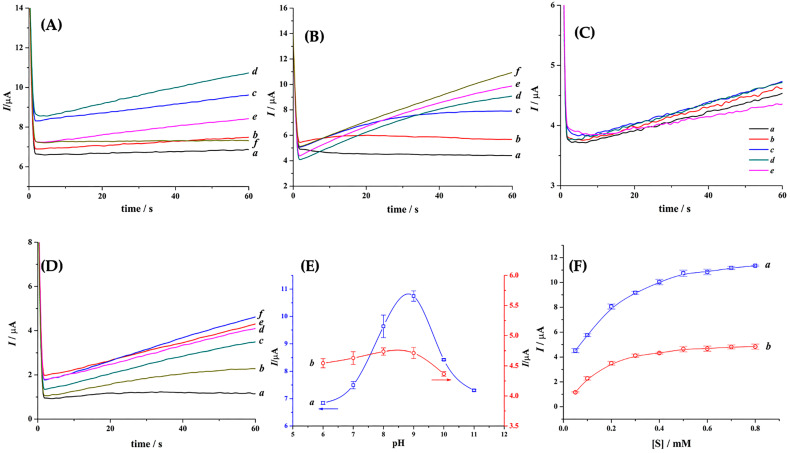
(**A**–**D**) Example of chronoamperograms obtained after background subtraction (**A**) in buffer solutions of pH (*a*) 6, (*b*) 7, (*c*) 8, (*d*) 9, (*e*) 10, and (*f*) 11, containing 0.50 mM Tos-Gly-Pro-Arg-ACP, 20 mM [Fe(EDTA)]^−1^, and 0.10 µM trypsin; (**B**) in 20 mM Tris (50 mM NaCl, 1.0 mM CaCl_2_) buffer (pH 8.8) solution containing 0.10 µM trypsin, 20 mM [Fe(EDTA)]^−1^, and (*a*) 0.05, (*b*) 0.10, (*c*) 0.20, (*d*) 0.30, (*e*) 0.40, and (*f*) 0.50 mM Tos-Gly-Pro-Arg-ACP. (**C**) in buffer solutions of pH (*a*) 6, (*b*) 7, (*c*) 8, (*d*) 9, (*e*) 10, containing 0.50 mM Tos-Gly-Pro-Arg-ACP, 20 mM [Fe(EDTA)]^−1^, and 1.0 µM thrombin; (**D**) in 20 mM Tris (50 mM NaCl, 1.0 mM CaCl_2_) buffer (pH 7.4) solution containing 1.0 µM thrombin, 20 mM [Fe(EDTA)]^−1^, and (*a*) 0.05, (*b*) 0.10, (*c*) 0.20, (*d*) 0.30, (*e*) 0.40, and (*f*) 0.50 mM Tos-Gly-Pro-Arg-ACP. (**E**,**F**) Variation of the mean current extracted from chronoamperograms at 60 s (error bar = ±1 SD; n = 3) as a function of (**E**) pH and (**F**) the substrate (Tos-Gly-Pro-Arg-ACP, [S]) concentration for (*a*) trypsin and (*b*) thrombin using the disposable co-facing gold electrodes and the conditions mentioned in (**A**–**D**). *E*_app_ = 0.4 V; T: 37 ± 0.2 °C.

The optimum working pH for each enzyme was evaluated (Figure 2A,C,E). The response of 0.10 µM trypsin in the presence of 0.50 mM Tos-Gly-Pro-Arg-ACP and 20 mM [Fe(EDTA)]^−1^, as well as the activity of 1.0 µM thrombin in the presence of 0.50 mM Tos-Gly-Pro-Arg-ACP, and 20 mM [Fe(EDTA)]^−1^ were investigated using 10 mM MES-HCl (0.50 M NaCl) buffer solution for pH 6; 10 mM Tris (0.50 M NaCl) buffer for pH 7 to 9; and finally, 10 mM borate (0.50 M NaCl) buffer for pH 10.

Figure 2A,E shows that trypsin has a maximum activity at ca. pH 8.8. However, thrombin shows a maximum activity between pH 6 and 9, with small but no statistically significant change observed in this range (Figure 2C,E). The breakpoint at about pH 9 corresponds to the pKa of arginine. The considerable activity differences observed between these two enzymes have important implications on the sensor development, as the pH will need to be regulated at 8.8 before every measurement that uses the trypsin enzyme. Nevertheless, it could operate at physiological pH (pH 7.4) when thrombin is used instead.

Figure 2 shows the variation of the current extracted from CA at 60 s obtained with 0.10 µM trypsin (Figure 2B,F), and 1.0 µM thrombin (Figure 2D,F) at the optimal pH conditions established in Figure 2E and with the Tos-Gly-Pro-Arg-ACP concentration varied from 0.05 to 0.80 mM. The enzyme concentration was selected, so a balance between the cost-effective production of the disposable strips and the test sensitivity was achieved. By carefully choosing this ratio, it is possible to extend this sensor detection range for lower and higher DAB concentrations. A systematic increase in the amperometric current at 60 s is observed as the [S] increases to ca. 0.20 mM for both enzymes. The current plateau after this [S] results from the enzymes arriving at their limiting catalytic values. It is important to note that, throughout this work, different enzyme (thrombin and trypsin) to substrate ratios were employed to validate the results and comments.

### 2.3. Dose-Response Curves of Dabigatran

Two data analysis methods were employed to compare the DAB quantification in the absence and presence of a calibration plot:

∆*k* vs. ln[DAB] or ∆*k* vs. [DAB]: *k* was obtained by fitting the pseudo linear part of the CA using Equation (1) [27].

(1) $$i\left(t\right)≅\frac{2zFADc_{red}^{*}}{L}\left(1+2exp\left(\frac{-4\pi^{2}Dt}{L^{2}}\right)\right)+\frac{2zFADkt}{L}+\frac{zFAkL}{2}\left(\frac{1}{3}-\frac{2}{\pi^{2}}exp\left(\frac{-4\pi^{2}Dt}{L^{2}}\right)\right)$$

where *D*, *A*, *F*, *z*, and *L* are the diffusion coefficient of the electroactive molecule, electrode area, the Faraday constant (96,485 C mol^−1^), the number of electrons exchanged, and the distance between the electrodes, respectively. This equation holds when two conditions are satisfied: (*i*) the generation of ACP should be pseudo-linear with time, and (*ii*) approximately 0.5 s should be allowed to elapse following the application of a potential so that the higher multiexponential terms have decayed more than 99% [27]. The mentioned fitting was performed using the following parameters: *A* = 0.11 cm^2^, *D*_ACP_ = 1.12 × 10^−5^ cm^2^ s^−1^, *z* = 2, and *L* = 95 μm. Then, a plot of ∆*k* vs. ln[DAB] or ∆*k* vs. [DAB] was generated to illustrate the correlation between the obtained information and the concentration used. This approach has a greater advantage because it does not require a calibration plot to get *c*_red_. As previously discussed, the effect of change in viscosity can be separated to obtain the value of *D*, *k*, and the concentration of the redox species [27]. In general, whenever ∆*k* was used in the plot, it should be considered that no calibration curve is required.

∆*I* vs. ln[DAB] or ∆*I* vs. [DAB]: By extracting current at 60 s from the corresponding CA, a plot of ∆*I* vs. ln[DAB] or ∆*I* vs. [DAB] gives the dose-response curve. However, in this scenario, just selecting current values at a particular time in CA, does not allow the separation of the effect of viscosity change on *D*, *k*, and *c*_red_. Therefore, a calibration curve is necessary to quantify DAB whenever ∆*I* is used for plotting.

The viability of this sensor to detect and quantify DAB in human plasma was also evaluated. As previously discussed, analysis of the transients using Equation (1) yields relatively constant *D* values when the open circuit potential is applied for 5 s [27]. A *D*_ACP_ = 1.16 ± 0.02 × 10^−5^ cm^2^ s^−1^ value was calculated by fitting Equation (1) to the experimental CA in ten-fold diluted plasma at 37 °C. A reaction rate constant, i.e., *k* = 7.06 × 10^−10^ M^−1^ s, was extracted from the fitting using the pseudo linear section of the experimental CA and the previously mentioned *D*_AC_ value.

**Figure 3:**
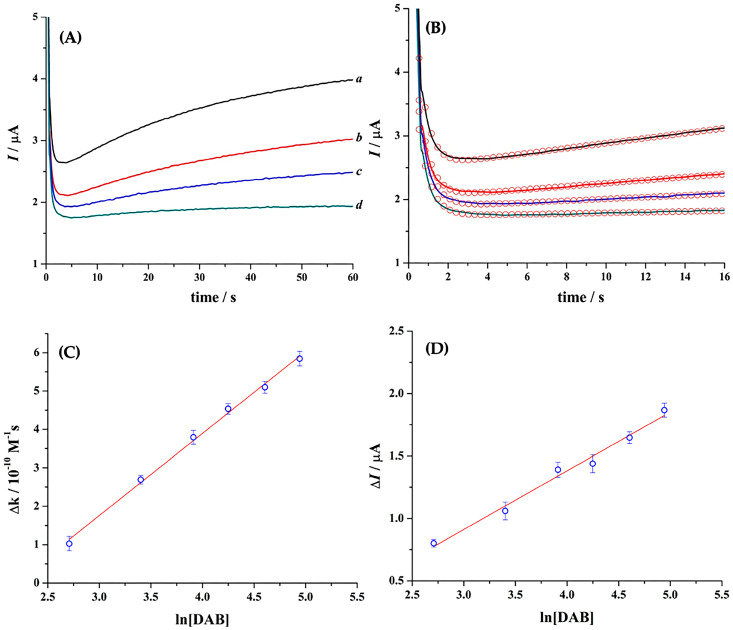
(**A**) Chronoamperograms after background subtraction of ten-fold diluted plasma in 20 mM Tris buffer (pH 7.4) solution containing 0.30 mM Tos-Gly-Pro-Arg-ACP, 20 mM [Fe(EDTA)]^−1^, 0.22 µM thrombin and (*a*) 0.0, (*b*) 15, (*c*) 30, and (*d*) 140 ng mL^−1^ DAB. (**B**) First 16 s of CA shown in A and the respective simulations (◯) using Equation (1). Simulation parameters: A = 0.11 cm^2^, $D_{ACP}$ = 1.16 × 10^−5^ cm^2^ s^−1^, z = 2, L = 95 µm. (**C**) Correlation between the change in the reaction rate constant (obtained from (B)) and the natural logarithm of the inhibitor concentration. (**D**) Correlation between the change in current magnitude extracted from the CAs at 60 s and the natural logarithm of DAB concentration. Error bars = ±1 SD; T = 22 ± 1 °C.

The detection of thrombin catalytic activity in ten-fold diluted serum has been reported previously [31]. Consequently, the same dilution will be used in this work to quantify DAB using the thrombin-based sensor. The following steps were applied: (*i*) a mixture containing ten-fold diluted plasma, 0.30 mM Tos-Gly-Pro-Arg-ACP, and 20 mM [Fe(EDTA)]^−1^ was loaded into the sensor, and CA was recorded. This serves as background current, which is later subtracted to each CA in this series; (*ii*) a ten-fold diluted plasma was mixed with 0.22 µM thrombin. Then, this mixture was added to a solution in 20 mM Tris buffer (pH 7.4) containing 0.30 mM Tos-Gly-Pro-Arg-ACP and 20 mM [Fe(EDTA)]^−1^. This final mixture was immediately injected into the sensor, and the corresponding CA was recorded; (*iii*) ten-fold diluted plasma spiked with different DAB concentrations was mixed with 0.22 µM thrombin. The resulting mixture was added to a 20 mM Tris buffer (pH 7.4) solution containing 0.30 mM Tos-Gly-Pro-Arg-ACP and 20 mM [Fe(EDTA)]^−1^. This final mixture was immediately injected into the sensor, and the CA was recorded (Figure 3A). These CAs, after background subtraction, show the effect of 0-140 ng mL^−1^ DAB concentration on the reaction rate constant, denoted by a decrease in the transient current with an increase in the DAB concentration. The rate constants were extracted from fitting Equation (1) to the respective pseudo-linear section of the experimental CAs (Figure 3B).

Figure 3C shows a linear relationship between ∆*k* vs. ln[DAB], with a linear regression equation ∆*k* = −4.65 × 10^−10^ + 2.13 × 10^−10^ ln[DAB]. The R^2^ for this plot was = 0.9959. A lower detection limit (LDL) of 9.6 ng mL^−1^ was obtained when a 3× standard deviation (SD) value was introduced to the linear regression equation and [DAB] calculated. Similarly, the lower quantification limit (LQL) of 11.5 ng mL^−1^ was extracted using a 10 × SD value in the same equation. These data show an excellent linear relation in the 11.5–140 ng mL^−1^ DAB concentration range.

Figure 3D represents a plot of change in current extracted from CA at 60 s, ∆*I* vs. ln[DAB]. The linear regression equation ∆*I* = −4.96 × 10^−7^ + 4.69 × 10^−7^ ln[DAB] was extracted from the fitting, showing an R^2^ of 0.9839. The LDL and LQL for this plot were 4.8 ng mL^−1^ and 15.6 ng mL^−1^, respectively. This graph shows good linearity in the 15.6–140 ng mL^−1^ DAB concentration range. Comparing Figure 3C,D, it is possible to see that both show excellent sensitivity for the point-of-care detection of DAB in diluted plasma samples. However, the ∆*k* vs. ln[DAB] is preferred as it provides [DAB] in less than 20 s without a calibration plot. Besides, this sensor can quantify ≤30 and ≤50 ng mL^−1^ DAB with high confidence. The quantification of <30 ng mL^−1^ DAB is critical to perform surgeries in cases of bleeding in emergency scenarios. Similarly, ≤50 ng mL^−1^ DAB is an upper range of quantification in patients suffering from ischemic stroke to initiate thrombolysis treatments [32].

**Figure 4:**
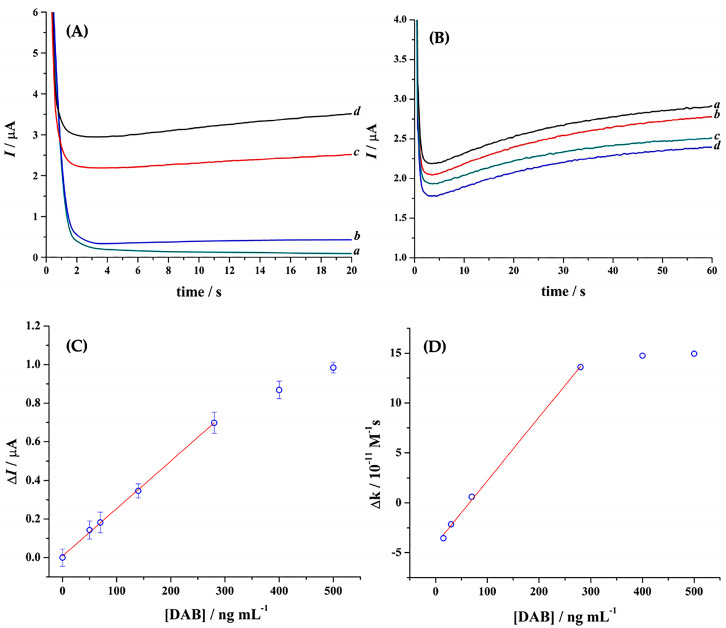
(**A**) Chronoamperograms obtained with the trypsin sensor to demonstrate the activity of this enzyme in different media: (*a*) Tris buffer (pH 8.8) solution; (*b*) plasma solution containing 0.1 µM trypsin, 0.07 mM Tos-Gly-Pro-Arg-ACP, and 20 mM [Fe(EDTA)]^−1^; (*c*) CA of the filtered plasma containing 0.10 µM trypsin, 0.07 mM Tos-Gly-Pro-Arg-ACP, and 20 mM [Fe(EDTA)]^−1^; and (*d*) CA obtained in a Tris buffer (pH 8.8) solution containing 0.10 µM trypsin, 0.07 mM Tos-Gly-Pro-Arg-ACP, and 20 mM [Fe(EDTA)]^−1^. *E*_app_ = 0.4 V; T: 37 ± 0.2 °C. (**B**) CA after background subtraction for filtered plasma samples containing 0.07 mM Tos-Gly-Pro-Arg-ACP, 20 mM [Fe(EDTA)]^−1^, 0.10 µM trypsin and (*a*) 0, (*b*) 70, (*c)* 280, and (*d*) 400 ng mL^−1^ DAB. (**C**,**D**) Correlation between the change in current magnitude (**C**) and the change in the reaction rate constant (**D**) extracted from the CAs presented in (B).

The viability of this sensor to detect and quantify [DAB] in human plasma using trypsin was also evaluated. Trypsin was added to a plasma sample, and its activity against Tos-Gly-Pro-Arg-ACP was monitored (Figure 4A). The resulting CA shows a significant reduction in the current magnitude. This may result from alpha-1 antitrypsin, a 52 kDa protein of the serpin family commonly present in the plasma [33,34]. This inhibition of the trypsin activity was observed even when the plasma was diluted 50, 70, and 90% in 20 mM Tris buffer (pH 8.8) solution. To test this hypothesis, the plasma was filtered using centrifugal filters of 10 kDa size cut-off before adding trypsin. Figure 4A shows that eliminating alpha-1 antitrypsin from plasma allows the recovery of approximately 75% of the trypsin activity in the Tris buffer.

The capacity of the assay to quantify DAB was then tested in filtered plasma (Figure 4B–D). The following procedure was used: (*i*) a background current was established with the filtered 7:3 plasma containing 0.10 µM trypsin and 20 mM [Fe(EDTA)]^−1^ in 20 mM Tris buffer (pH 8.8); (*ii*) a filtered plasma was then mixed with 0.10 µM and a solution containing 0.07 mM Tos-Gly-Pro-Arg-ACP and 20 mM [Fe(EDTA)]^−1^ in 20 mM Tris buffer (pH 8.8). This 7:3 filtered plasma:buffer mixture was injected into the sensor immediately, and the corresponding CA was recorded; then (*iii*) the plasma was spiked with different DAB concentrations and filtered as previously described. This was then mixed with 0.10 µM trypsin and a solution containing 0.07 mM Tos-Gly-Pro-Arg-ACP and 20 mM [Fe(EDTA)]^−1^ in 20 mM Tris buffer (pH 8.8) solution. This solution was injected into the sensor, and the corresponding CAs were recorded.

Figure 4B illustrates different CA after background subtraction of 0–400 ng mL^−1^ DAB in 7:3 filtered plasma. A decrease in transient current for an increase in DAB concentration was observed. Figure 4C shows a plot of change in current at 60 s vs. DAB concentration in the range of 0–500 ng mL^−1^. The Δ*I* vs. [DAB] follows the linear regression equation Δ*I* = 7.91 × 10^−9^ + 2.46 × 10^−9^ [DAB] with R^2^= 0.9988 up to 280 ng mL^−1^ under the current trypsin to substrate ratio. The LDL and LQL were 50.7 and 177 ng mL^−1^, respectively.

Figure 4D illustrates the plot of change in the rate constant, Δ*k* vs. [DAB], within the range of 0–500 ng mL^−1^. The *Δk* values were obtained by fitting the pseudo linear section of various CA (Figure 4B) using Equation (1). The plot of Δ*k* vs. [DAB] returned poor linearity and negative values at a low concentration of DAB.

This poor performance, which is related to the large standard deviation obtained, severely limits the application of the trypsin-based sensor. A possible explanation for these results may be that during the DAB-spiked plasma filtration process, DAB interacted with the filtration medium or plasma proteins, significantly reducing its availability within the filtrate.

Based on the differences between the trypsin and thrombin sensors, the thrombin sensor has the potential to be used in point-of-care anticoagulation therapy due to its relatively wide pH working range and minimum plasma manipulation required. Another essential feature of this sensor is the excellent response time needed to run a single measurement, which is less than 20 s, and a calibration-free setup if the change in the rate constant is used. In addition, it requires 7.0 µL of the sample, which is one of the main requirements of any point-of-care diagnostic system.

Assays for reproducibility were conducted employing repetitive standards solutions in plasma diluted by a factor of ten (n = 3) using a 20 mM tris buffer (pH 7.4) solution composed of 0.22 µM thrombin, 0.3 mM Tos-Gly-Pro-Arg-ACP, and 20 mM [Fe(EDTA)]^−1^ along with varying concentrations of DAB (Table 1). The resultant data exhibited low values of the standard error of the mean (SEM), demonstrating the excellent precision of the proposed DAB sensor. Moreover, the table illustrates that the actual quantities of DAB recovered closely mirrored the quantities of DAB initially added, thus attesting to the high accuracy of the constructed sensor. Besides, as the background subtraction step eliminates the interference of all redox active molecules present in plasma, the sensor offers high selectivity.

## 3. Materials and Methods

Tris(hydroxymethyl)aminomethane (Tris, ≥99%), calcium chloride dihydrate, sodium chloride, magnesium chloride, thrombin from bovine plasma, trypsin from bovine pancreas, phosphate buffer saline (PBS) tablets, ethylenediaminetetraacetic acid ferric sodium salt (Na[FeEDTA], 99%) and Tween-20 were purchased from Sigma-Aldrich and used without further purification. The substrate Tos-Gly-Pro-Arg-ACP was purchased from DSM-Nutritional Products Ltd. Branch Pentapharm (Aesch, Switzerland) and reconstituted in Milli-Q water (18.2 MΩ cm^−1^) to a 5.0 mM concentration. This solution was prepared daily and stored at 4 °C when not in use. Nanosep devices of 30 kDa molecular weight cut-off were purchased from Pall Corporation (New York, NY, USA). Dabigatran was purchased from Toronto Research Chemicals (Toronto, ON, Canada) and used without further purification. Although all concentrations in this work are reported in molar concentrations, the DAB concentration will be reported in mg mL^−1^. A 1.0 mg mL^−1^ DAB in 0.10 mM hydrochloric acid stock solution was prepared daily and stored at 4 °C when not in use. Lyophilised pooled plasma was purchased from Sigma-Aldrich and reconstituted using Milli-Q as per instructions (Sigma-Aldrich, Castle Hill, Australia). This solution was kept at room temperature for about 30 min to stabilise and finally stored at 4 °C when not used.

Thrombin solution was prepared to 100 µM concentration using 10 mM Tris-HCl buffer pH 7.0, 0.50 M NaCl. A 1.0 µM trypsin solution was prepared using 10 mM Tris-HCl buffer, pH 8.8, containing 0.50 M NaCl, 0.05 M CaCl_2_, 0.02 M MgCl_2_ and 0.01% Tween-20. Both these enzyme solutions were stored at −20 °C when not in use. From the above stock solutions, respective enzyme aliquots were prepared just before the experiments.

Electrochemical measurements: All electrochemical measurements were carried out using a WaveNow potentiostat (Pine Research, Durham, NC, USA). Disposable co-facing gold electrodes (two electrodes system, Scheme 1) were supplied by Universal Biosensors Inc. (Rowville, Australia). The characteristics of this sensor were previously described [27] and consists of two 10 to 15 nm gold sputtered on Melinex sheets (Eastman Performance Films, Martinsville, VA, USA) separated by a 95 µm thick double-sided adhesive PET spacer (Adhesive research INC, Glen Rock, PA, USA), where one electrode acts as the working electrode and the other as both the counter and the pseudo-reference electrode. The disposable sensors were heated to 37 ± 0.2 °C on an external digitally controlled heating bar and maintained under this condition until the end of the assay. A potential of 0.40 V was applied to the working electrode to collect all CAs.

The assays in buffer solutions were performed using a fixed sample volume of 7.0 µL, which is large enough to fill the electrode chamber. To this end, 2.0 µL of 0.26 µM trypsin was mixed with 4.13 µL of Tris buffer and incubated at room temperature for 10 min. These samples were spiked with different DAB concentrations to cover the 0–600 ng mL^−1^ range. Then, 0.88 µL of 2.0 mM substrate containing 20 mM of [Fe(EDTA)]^−1^ was added to this mixture and immediately loaded into the test strips.

The assay in plasma using trypsin was performed by spiking this solution with different DAB concentrations to cover the range of 0–600 ng mL^−1^ and then centrifuged using Nanosep devices for about 30 min at 12,000× *g*. Finally, 3.60 µL of filtered plasma was added to 2.0 µL of 0.32 µM trypsin and 1.40 µL of 2.50 mM substrate containing 20 mM of [Fe(EDTA)]^−1^. This mixture was immediately loaded into the test strip, and the respective CA was recorded.

Similarly, the assay in plasma using thrombin also requires spiking the solution with different DAB concentrations to cover the range of 0–500 ng mL^−1^. The plasma solution was then diluted ten-fold with 10 mM Tris buffer pH-7.4 (0.50 M NaCl). Then, 3.60 µL of diluted plasma was added to 2.0 µL of 3.50 µM thrombin and 1.40 µL of 3.0 mM substrate containing 20 mM of [Fe(EDTA)]^−1^. This mixture was immediately loaded into the test strip, and the respective CA was recorded.

## 4. Conclusions

Two disposable sensors were constructed and optimised to detect the anticoagulant drug DAB using novel co-facing disposable electrodes that separate the effects due to viscosity and concentration, allowing the improved quantitation of the electroactive mediator concentration.

In the case of the trypsin-based sensor, lower concentrations of DAB ≤30–50 ng mL^−1^ could not be quantified. Non-specific interactions of trypsin with plasma components and low DAB affinity with trypsin were the primary reasons for this lack of sensitivity. For example, when plotting Δ*I* vs. [DAB], this sensor could detect 50.7 ng mL^−1^ DAB and quantify it in the 177–280 ng mL^−1^ range. Consequently, the trypsin-based sensor does not fulfil the requirements for a point-of-care anticoagulation diagnostic tool due to the pH sensitivity (pH limited to 8.8) and prolonged plasma filtration required before the DAB concentration is measured.

Meanwhile, the thrombin-based sensor performed well when diluted plasma samples were used to quantify DAB. This sensor can detect ≥9.6 ng mL^−1^ DAB and quantify 11.5–140 ng mL^−1^ when Δ*k* vs ln[DAB] was used. Similarly, an LDL and LQL of 4.8 and 15.6 ng mL^−1^ DAB, respectively, were achieved when the Δ*I* vs. ln[DAB] plot was considered. These sensors covered the therapeutic range and clinically significant concentrations. This is particularly important in an emergency centre where concentrations ≤ 30–50 ng mL^−1^ DAB are deemed safe for emergency surgeries for people under DAB therapy. If the *k* values are used, a high-performance calibration-free sensor can be achieved. Its extensive pH stability range, the possibility of working at physiological pH, and its low cost and fast turnaround response make the thrombin-based sensor a suitable DAB-specific point-of-care test to measure the drug concentration in particular conditions of massive bleeding or emergency surgeries.

## Figures and Tables

**Table 1:** Accuracy and precision data (n = 3) for DAB measurement in tenfold diluted plasma using the thrombin-based sensor.

DAB added (ng mL^−1^)	20.0	30.0	40.0	50.0
Mean DAB found (ng mL^−1^)	20.8	28.5	41.7	48.3
Recovery (%)	104	95.0	104	96.6
SEM (%)	1.4	0.9	1.7	0.4

SEM = standard error of mean.

## Data Availability

Data will be made available on request.
